# Supplementary material for: Investment Opportunities for mRNA Technology in Low- and Middle-Income Countries: Key Findings and Future Perspectives
Source: Vaccines (Basel). 2025 Jan 23;13(2):112. doi: 10.3390/vaccines13020112 (PMC11861337; doi:10.3390/vaccines13020112)
Supplement: Supplementary file 1 [file vaccines-13-00112-s001.zip › vaccines-3407999-supplementary.pdf]

Supplementary files

Table S1: Description of the meeting participant institutions.

Participant Type	Institution
Academic Institution	Johns Hopkins Bloomberg School of Public Health
Biotechnology Company*	Afrigen Biologics & Vaccines #
Pharmaceutical Company*	Biofarma
	BioGeneric Pharma S.A.E.
	Biological E Ltd
	Darnytsia Pharmaceutical Company
	Institut Pasteur de Dakar
	Institut Pasteur de Tunise
	Instituto de Tecnologia em Imunobiológicos (Bio-Manguinhos)
	Kenya BioVax Institute
	National Institutes of Health Pakistan
	Polyvac
	Sinergium Biotech
	The Biovac Institute
	Torlak institute
Multilateral Development Bank	African Development Bank
	Asian Development Bank
	Asian Infrastructure Investment Bank
	Inter-American Development Bank
	International Finance Corporation
	Islamic Development Bank
Intergovernmental Organization	Pan American Health Organization
	World Health Organization
International Organization	the Medicines Patents Pool

Table S2: WHO/MPP mRNA Tech Transfer Programme: Investment Opportunities meeting agenda.

**Time**	**Topic**	**Comments**
Introductory session	Welcome, and the introduction of all participants.<br><br>*Claudia Nannei, Senior Programme Manager, WHO*	WHO and PAHO.

	Judit Rius Sanjuan, Director, Department on Innovation and Access to Health Technologies, PAHO	
	Overview of the mRNA TT programme and its current activities.<br><br>*Matthias Helble, Scientist, Research for Health Department, Science Division, WHO*<br><br>*Claudia Nannei, Senior Programme Manager & Ariane Abreu, consultant, Access to Medicines and Health Products Division, WHO*	Overview of the programme and report back from latest activities (consortia, gaps and needs assessments at partners).
	Setting the scene.<br><br>*Zeynep Kantur Ozenci, Global Sector Manager, Health & Education, IFC*	Outcomes of IFC-WHO event on 16 April, and high-level views on the role of MDBs in building capacities for new health technologies in LMICs.
Session 1	Investment models of multilateral development banks for local manufacturing of health products.<br><br>*Moderator: Ike James, Technology Transfer Lead, MPP*<br><br>*Panelists:*<br>• *Hun Kim, Director General, AIIB (remote)*<br>• *Eduardo Banzon, Principal Health Specialist, ADB (remote)*<br>• *Rodrigo Navas, Director, IDB*<br>• *Zeynep Kantur Ozenci, Global Sector Manager, Health & Education, IFC*	Presentation of instruments to fund research, development, and manufacturing of health products in LMICs.
Session 2	Presentation of the CAPEX-OPEX model and estimated capacity to contribute to a pandemic response.<br><br>*Martin Nicholson, IVB, WHO (remote)*	Estimates of costs to set up and run an mRNA facility with 3 scenarios;<br><br>Estimates of potential contribution to a global supply of vaccines during a

		pandemic response by the mRNA partners
Session 3	Building the ecosystem for the development of new mRNA-based products in LMICs.<br><br>Moderator: Anthony So, Professor, Johns Hopkins University<br>Panelists:<br>• *Ammar Abdo Ahmed, IsDB (remote)*<br>• *Chaouki Benabdessalem, Member of mRNA TASK force, Institut Pasteur Tunis*<br>• *Mauricio Zuma Medeiros, Director, Bio-Manguinhos*<br>• *Ousmane Fall, Director, AfDB*<br>• *Michael Karl Schunk, IFC (tbc)*<br>• *Petro Terblanche, Managing Director, Afrigen*	To discuss the issues linked to research and development of new mRNA products and how MDB and partners can contribute to creating a sustainable investment
Session 4	Addressing the needs of a nascent mRNA industry in LMICs.<br><br>Moderator: Ariane Lopes de Abreu, WHO<br>Panelists:<br>• *Fernando Lobos, Business Development Director, Sinergium*<br>• *Ken Osei, IFC/World Bank*<br>• *Luka Dragačević, Managing Director, Torlak*<br>• *Michael Lusiola, Managing Director, Biovax*<br>• *Morena Makhoana, CEO, Biovac*	To discuss from a policy and financial support perspective the needs of new industries being created in LMICs (funding sources, market access, procurement practices, international competition, etc.).
Session 5	Open discussion: Can we envisage new models for LMIC manufacturers to make access to financing opportunities easier?	Challenging the status quo to finance the expansion of biomanufacturing

	Moderator: Ken Osei, IFC/World Bank<br><br>*Questions for open discussion will be distributed at the beginning of the meeting to all participants.*	capacities in LMICs and discuss innovative ideas.
Closing remarks	Conclusions and next steps by WHO and PAHO<br><br>*Claudia Nannei, Senior Programme Manager, MHP/WHO, and Francisco Caccavo, Medicines and Health Technologies Unit, PAHO*	WHO and PAHO.
